# Effect of Pneumococcal Conjugate Vaccination on Serotype-Specific Carriage and Invasive Disease in England: A Cross-Sectional Study

**DOI:** 10.1371/journal.pmed.1001017

**Published:** 2011-04-05

**Authors:** Stefan Flasche, Albert Jan Van Hoek, Elizabeth Sheasby, Pauline Waight, Nick Andrews, Carmen Sheppard, Robert George, Elizabeth Miller

**Affiliations:** 1 Immunisation, Hepatitis and Blood Safety Department, Health Protection Agency, London, United Kingdom; 2 Department of Mathematics and Statistics, Strathclyde University, Glasgow, United Kingdom; 3 Respiratory & Systemic Infection Laboratory (RSIL), Health Protection Agency, London, United Kingdom; Emory University, United States of America

## Abstract

**Background:**

We investigated the effect of the 7-valent pneumococcal conjugate vaccine (PCV7) programme in England on serotype-specific carriage and invasive disease to help understand its role in serotype replacement and predict the impact of higher valency vaccines.

**Methods and Findings:**

Nasopharyngeal swabs were taken from children <5 y old and family members (*n* = 400) 2 y after introduction of PCV7 into routine immunization programs. Proportions carrying *Streptococcus pneumoniae* and serotype distribution among carried isolates were compared with a similar population prior to PCV7 introduction. Serotype-specific case∶carrier ratios (CCRs) were estimated using national data on invasive disease. In vaccinated children and their contacts vaccine-type (VT) carriage decreased, but was offset by an increase in non-VT carriage, with no significant overall change in carriage prevalence, odds ratio 1.06 (95% confidence interval 0.76–1.49). The lower CCRs of the replacing serotypes resulted in a net reduction in invasive disease in children. The additional serotypes covered by higher valency vaccines had low carriage but high disease prevalence. Serotype 11C emerged as predominant in carriage but caused no invasive disease whereas 8, 12F, and 22F emerged in disease but had very low carriage prevalence.

**Conclusion:**

Because the additional serotypes included in PCV10/13 have high CCRs but low carriage prevalence, vaccinating against them is likely to significantly reduce invasive disease with less risk of serotype replacement. However, a few serotypes with high CCRs could mitigate the benefits of higher valency vaccines. Assessment of the effect of PCV on carriage as well as invasive disease should be part of enhanced surveillance activities for PCVs.

*Please see later in the article for the Editors' Summary*

## Abstract 2

A cross sectional study by Stefan Flasche and coworkers document the serotype replacement of *Streptococcus pneumoniae* that has occurred in England since the introduction of PCV7 vaccination.

## Editors' Summary

**Background:**

Pneumococcal diseases—major causes of illness and death in children and adults worldwide—are caused by *Streptococcus pneumoniae*, a bacterium that often colonizes the nasopharynx (the area of the throat behind the nose). Carriage of *S. pneumoniae* bacteria does not necessarily cause disease. However, these bacteria can cause local, noninvasive diseases such as ear infections and sinusitis and, more rarely, they can spread into the lungs, the bloodstream, or the covering of the brain, where they cause pneumonia, septicemia, and meningitis, respectively. Although these invasive pneumococcal diseases (IPDs) can be successfully treated if administered early, they can be fatal. Consequently, it is better to protect people against IPDs through vaccination than risk infection. Vaccination primes the immune system to recognize and attack disease-causing organisms (pathogens) rapidly and effectively by exposing it to weakened or dead pathogens or to pathogen molecules (antigens) that it recognizes as foreign.

**Why Was This Study Done?:**

There are more than 90 *S. pneumoniae* variants or “serotypes” characterized by different polysaccharide (complex sugar) coats, which trigger the immune response against *S. pneumoniae* and determine each serotype's propensity to cause IPD. The pneumococcal conjugate vaccine PCV7 contains polysaccharides (linked to a protein carrier) from the seven serotypes mainly responsible for IPD in the US in 2000 when routine childhood PCV7 vaccination was introduced in that country. PCV7 prevents both IPD caused by the serotypes it contains and carriage of these serotypes, which means that, after vaccination, previously uncommon, nonvaccine serotypes can colonize the nasopharynx. If these serotypes have a high invasiveness potential, then “serotype replacement” could reduce the benefits of vaccination. In this cross-sectional study (a study that investigates the relationship between a disease and an intervention in a population at one time point), the researchers investigate the effect of the UK PCV7 vaccination program (which began in 2006) on serotype-specific carriage and IPD in England to understand the role of PCV7 in serotype replacement and to predict the likely impact of vaccines containing additional serotypes (higher valency vaccines).

**What Did the Researchers Do and Find?:**

The researchers examined nasopharyngeal swabs taken from PCV7-vaccinated children and their families for *S. pneumoniae*, determined the serotype of any bacteria they found, and compared the proportion of people carrying *S. pneumoniae* (carrier prevalence) and the distribution of serotypes in this study population and in a similar population that was studied in 2000/2001, before the PCV vaccination program began. Overall, there was no statistically significant change in carrier prevalence, but carriage of vaccine serotypes decreased in vaccinated children and their contacts whereas carriage of nonvaccine serotypes increased. The serotype-specific case-to-carrier ratios (CCRs; a measure of serotype invasiveness that was estimated using national IPD data) of the replacing serotypes were generally lower than those of the original serotypes, which resulted in a net reduction in IPD in children. Moreover, before PCV7 vaccination began, PCV7-included serotypes were responsible for similar proportions of pneumococcal carriage and disease; afterwards, the additional serotypes present in the higher valency vaccines PVC10 and PVC13 were responsible for a higher proportion of disease than carriage. Finally, three serotypes not present in the higher valency vaccines with outstandingly high CCRs (high invasiveness potential) are identified.

**What Do These Findings Mean?:**

These findings document the serotype replacement of *S. pneumoniae* that has occurred in England since the introduction of PCV7 vaccination and highlight the importance of assessing the effects of pneumococcal vaccines on carriage as well as on IPDs. Because the additional serotypes included in PCV10 and PCV13 have high CCRs but low carriage prevalence and because most of the potential replacement serotypes have low CCRs, these findings suggest that the introduction of higher valency vaccines should further reduce the occurrence of invasive disease with limited risk of additional serotype replacement. However, the emergence of a few serotypes that have high CCRs but are not included in PCV10 and PCV13 might mitigate the benefits of higher valency vaccines. In other words, although the recent introduction of PCV13 into UK vaccination schedules is likely to have an incremental benefit on the reduction of IPD compared to PCV7, this benefit might be offset by increases in the carriage of some high CCR serotypes. These serotypes should be considered for inclusion in future vaccines.

**Additional Information:**

Please access these Web sites via the online version of this summary at http://dx.doi.org/10.1371/journal.pmed.1001017.

- The US Centers for Disease Control and Prevention provides information for patients and health professionals on all aspects of pneumococcal disease and pneumococcal vaccination
- The US National Foundation for Infectious Diseases has a fact sheet on pneumococcal diseases
- The UK Health Protection Agency provides information on pneumococcal disease and on pneumococcal vaccines
- The World Health Organization also provides information on pneumococcal vaccines
- MedlinePlus has links to further information about pneumococcal infections (in English and Spanish)

## Introduction

*Streptococcus pneumoniae* is a bacterium that frequently colonises the human nasopharynx. Apart from disease outcomes such as sinusitis, otitis media, and community-acquired pneumonia, which result from direct spread from the nasopharynx, the pneumococcus can invade the bloodstream and cause septicaemia, meningitis, and invasive pneumonia. Most carriage episodes, however, do not result in either local or systemic disease. It is believed that the propensity to cause invasive disease in healthy individuals—termed invasiveness—is largely determined by the characteristics of the pneumococcus' polysaccharide capsule, although the explicit underlying mechanisms are yet to be identified [1],[2]. On the basis of the immune response to differences in capsular polysaccharide structure, more than 90 serotypes causing invasive disease have been described [3].

A pneumococcal conjugate vaccine (PCV7) that induces anticapsular antibodies against the seven serotypes, which at that time were responsible for most of the pneumococcal invasive disease in the United States (US), was introduced into the US childhood immunisation schedule in 2000 and the majority of the developed world subsequently. Since PCV7 is protective against invasive pneumococcal disease (IPD) [4] and carriage [5],[6], the assumption of protection of the unvaccinated against vaccine type (VT) IPD through herd immunity played a major role in evaluating the likely impact and cost-effectiveness of vaccination [7]. Prevention of VT carriage, however, creates a potential ecological niche in the nasopharynx for previously less prevalent serotypes to emerge (replacement).

The extent to which the benefits of herd immunity will be offset by serotype replacement is hard to predict [8] and may vary by country depending on local factors such as differences in serotype distribution before vaccination and the population demography. Hence, there is a need for enhanced surveillance to evaluate the effect of vaccination in different epidemiological settings. Most surveillance systems focus on IPD and have shown large reductions in the numbers of VT cases in the targeted age groups, irrespective of vaccine schedule [9]–[11]. However, differences were observed in the indirect effect (i.e., the degree of induced herd immunity and the level of non–vaccine-type [NVT] replacement), the reasons for which remain unclear but may include vaccine coverage, time since introduction of PCV, and sensitivity of the reporting system [12].

Monitoring disease outcomes provides little insight into the underlying mechanisms that determine herd immunity and serotype replacement. For this, carriage data are essential. Carriage studies in children from Massachusetts and Norway suggest full replacement of pneumococcus in carriage after PCV7 introduction [13],[14]. The implications of changes in serotype-specific carriage prevalence for expression as IPD will, however, depend on the invasiveness of individual serotypes, which is reflected by the case∶carrier ratio (CCR). Invasiveness has only been studied in one of these settings and was restricted to children [15],[16]. Improving our understanding of this relationship, largely determined by the invasiveness potential of the replacing NVT organisms, is essential to understanding the effect of PCV7 in different epidemiological settings.

In September 2006, PCV7 was introduced into the immunisation schedule in the United Kingdom as a 2/4/13-month routine schedule with a catch-up for children up to 2 y of age. Information on carriage in England prior to PCV7 introduction is available from a longitudinal study conducted in 2001/2002 in index children and their household members. We report here the results of a cross-sectional carriage study conducted in a demographically similar population in 2008/2009. We compare our post-PCV7 findings with the pre-PCV7 baseline both for carriage and IPD to help understand the serotype-specific effects of PCV7 on both carriage and IPD and use this analysis to predict the potential impact of higher valency conjugate vaccines on herd immunity and replacement disease.

## Methods

### Study Population

Children born since 4 September 2004 and thus eligible for routine or catch-up PCV were recruited along with family members from general practices in Hertfordshire and Gloucestershire. Exclusion criteria were: moderate to severe disability, cerebral palsy, neurological disorders affecting swallowing, ear, nose, and throat disorders affecting the anatomy of the ear, or immunosuppression. The NHS National Research Ethics Service approved the study protocol. Written informed consent was obtained from adult study participants and from a parent/guardian of study children prior to enrolment. Information was collected on participants' age, gender, household size, number of smokers in household, recent antibiotic treatment, hours in day-care and PCV7 vaccination history.

To compare to prevaccination carriage in England, we used the results from a longitudinal study carried out in 2001/2002 in families attending the same general practices in Hertfordshire in which swabs were taken each month over a 10-mo period [17]. At that time, serotype 6C could not be distinguished from 6A, but in 2009, 19 of the 122 serotype 6As from the earlier study were randomly retested, six of which were found to be 6C. We have assumed that this proportion (32%) holds for the rest of the 6A carriage isolates from the 2001/2002 study.

### Specimen Collection and Testing

Nasopharyngeal swabs (calcium-alginate) were taken between April 2008 and November 2009 by trained nurses and placed directly in STGG broth. Samples collected at Hertfordshire were sent by same day courier to the Respiratory and Systemic Infection Laboratory at the Centre for Infections (RSIL). They were stored overnight in at 2–8°C and frozen the next morning at −80°C. Samples collected at Gloucestershire were stored locally at the Gloucester Vaccine Evaluation Unit at −80°C and transferred to RSIL in batches on dry ice. On receipt the batches were stored at −80°C. The sample then was thawed, vortexed, and 50 µl STGG broth was placed onto each of Columbia blood agar plate (HPA media services) with optochin disc (MAST) and Streptococcus-selective Columbia blood agar plate (HPA media services) and streaked out. The plates were incubated overnight at 35°C with 5% CO_2_. Any colonies resembling pneumococcus were subjected to normal identification methods and serotyped using the standard laboratory protocol [18].

### Statistical Analysis

Descriptive data analysis was performed in R 2.11.0 and Generalized Estimating Equations (GEEs) models were analysed with STATA 10.1. Exact binomial 95% confidence intervals (CIs) were obtained for carriage rates in 2008/2009 by age group (<5, 5–20,>20 y). To account for longitudinal design in the 2001/2002 study, we computed these carriage rates using a GEE model with exchangeable correlation structure. To determine the significance of changes in carriage for individual serotypes between 2001/2002 and 2008/2009, a Fisher exact test was used because of small numbers. When comparing overall carriage as well as vaccine and NVT carriage between periods, this comparison took account of the longitudinal design of the 2001/2002 along with other covariates by using a GEE model with exchangeable correlation structure and factors for study period, age in years, gender, whether the household has a smoker, and the number of children and adults in the household. For comparability with previously reported changes in carriage, the data were stratified into two age groups (<5 and ≥5 y).

For calculating the CCR the numbers of each serotype were extracted from the national surveillance database for England and Wales [19] for the epidemiological years 2001/2002 and 2008/2009 and related to data from the carriage studies conducted in the same years (Table S1). CCRs were calculated using serotype-specific carriage prevalence as denominator. Ages younger than 60 y were combined in both the IPD and carriage datasets. 95% CIs were calculated on the basis of the 95% CIs of the serotype-specific carriage prevalence assuming the national incidence data on IPD to be complete and not based on a population sample [19]. For serotypes with estimates in both datasets, Spearman's rank test was used to estimate the correlation of our estimates and those obtained by Sleeman and colleagues from a paediatric pre-PCV7 carriage dataset in one region of England corrected for duration of carriage [20].

Simpson's index for diversity was calculated to assess the change in diversity in the bacterial population following vaccination [21]. Ranked serotype distribution was compared to the prevaccination distribution and CIs were obtained using the methods described by Hanage and colleagues [22]. To ensure that only a single isolate per carriage episode was included we excluded consecutive swabs with the same serotype (this included swabs of more than one sample interval apart if the individual was not sampled in between) in the 2001/2002 study on the assumption that it was carriage persisting from the previous month.

## Results

400 individuals were enrolled between 24 April 2008 and 9 November 2009. One participant withdrew before being swabbed and in 17 individuals swabbing had to be aborted early; these 18 participants were excluded from further analyses. The demographic features of the remaining 382 participants were similar to the participants in the 2001/2002 study, apart from the proportion of households with at least one smoker, which was lower in the more recent study (Table 1). Of 180 children eligible for catch-up or infant vaccination only four were unvaccinated.

**Table 1:** Overview of numbers of participants recruited, their demographic features, and household structures in the 2001/2002 and 2008/2009 carriage studies.

Participants, Demographics, and Household Structures	2001/2002	2008/2009
*n* Participants	488	382
*n* Swabs taken	3,868	382
*n* Participants <5 y (%)	180 (37)	192 (50)
*n* Participants 5–20 y (%)	71 (15)	57 (15)
*n* Participants >20 y (%)	237 (49)	133 (35)
*n* Proportion female	53.0%	56.4%
*n* HH	121	146
Median HH size (range)	4 (2–7)	4 (3–7)
Median *n* adults in HH (range)	2 (1–5)	2 (1–5)
Median *n* children in HH (range)	1 (1–3)	2 (1–4)
Proportion of smoke-free HH	66.9%	81.0%

HH, household.

A pneumococcus was grown from 127 of the 382 (33.2%) swabs and a serotype determined in 123 (97%). The most prevalent serotypes were 19A (10), 23B (9), 11C (8), 15B (8), 21 (8), and 6C (8). Compared to prevaccination levels, we found a significant reduction in carriage of VTs 6B, 14, 19F, 23F, and 6A. For the remaining PCV7 types no carriage episodes of serotypes 4 and 9V were found postvaccination, but prevaccination levels were too low to detect any significant change. VT 18C was identified in three out of 382 (0.79%) swabs in 2008/2009 and in 25 out of 3,868 (0.64%) in the 2001/2002 study. NVTs 33F, 7F, 10A, 34, 15B, 31, 21, 3, 19A, 15C, and 23A significantly increased (*p*<0.05) in carriage with odds of 40.9, 30.8, 20.4, 20.3, 16.5, 10.2, 8.2, 6.2, 4.5, 3.6, and 3.6, respectively. A significant increase was also found in serotypes 23B, 11C, 11B, 24F, and 33A, which were only detected in the postvaccination study.

The proportion of swabs with VT and NVT serotypes according to age group in both studies is shown in Table 2. The odds ratio of VT and NVT carriage postvaccination compared to prevaccination using the GEE with binary outcome was estimated to be 0.07 95% CI (0.03–0.16) and 4.40 95% CI (3.06–6.33), respectively, with, no significant effect on overall carriage: 1.06 95% CI (0.76–1.49) (Table 3). When applying the same models to individuals younger than 5 y only, we found similar patterns. In individuals aged 5 y or older, we detected evidence for herd immunity and full serotype replacement as well (odds ratio [OR] 0.31 95% CI (0.04–2.49), 5.16 95% CI (1.95–13.66), respectively), although the reduction in VT carriage was not significant.

**Table 2:** Number of positive VT, NVT, and All (including nontypeable) carriage isolates in 2008/2009.

Age	Cases	NVT	VT	All
<5 y	Cases 2008/2009 (*n* = 192)	87	7	98
	Proportion 2008/2009	45.3% (38.5–52.6)	3.6% (1.0–6.2)	51.0% (43.8–58.3)
	Proportion 2001/2002^a^	15.3% (12.7–18.3)	31.9% (28.1–36.1)	48.4% (44.1–52.7)
5–20 y	Cases 2008/2009 (*n* = 57)	15	0	16
	Proportion 2008/2009	26.3% (15.8–38.6)	0% (0–6.4)	28.1% (17.5–40.4)
	Proportion 2001/2002^a^	9.1% (6.3–12.8)	9.9% (7.3–13.3)	20.6% (16.1–26.1)
>20 y	Cases 2008/2009 (*n* = 133)	10	3	13
	Proportion 2008/2009	7.5% (3–12)	2.3% (0–5.3)	9.8% (5.3–15)
	Proportion 2001/2002^a^	3.3% (2.4–4.8)	4.1% (3.0–5.5)	7.6% (6.2–9.5)
All	Cases 2008/2009 (*n* = 382)	112	10	127
	Proportion 2008/2009	29.3% (24.9–34)	2.6% (1–4.5)	31.9% (27.2–36.6)
	Proportion 2001/2002^a^	8.5% (7.2–9.9)	15.2% (13.2–17.4)	24.4% (21.9–27.1)

a The proportion for 2001/2002 was calculated accounting for multiple testing of the participants.

**Table 3:** Odds ratios for comparing 2001/2002 to 2008/2009 carriage using GEE.

Participants	<5 y		>5 y		All	
VT	0.06 (0.03–0.16)	abg ***	0.31 (0.04–2.49)	a***	0.07 (0.03–0.16)	aeg ***, b**
NVT	4.25 (2.81–6.43)	c*,g***	5.16 (1.95–13.66)	ag**	4.40 (3.06–6.33)	ag***, bc*
All	1.03 (0.70–1.51)	ab***, e*	2.46 (1.04–5.83)	a***, g*	1.06 (0.76–1.49)	ab***,e**

Key for significant fixed effects: a, age; b, antibiotic treatment; c, smoking; d, gender; e, adults in household; f, children in household; g, study period.

Significance codes:

*≤0.05;

**≤0.01;

***≤0.001.

**Figure 1:**
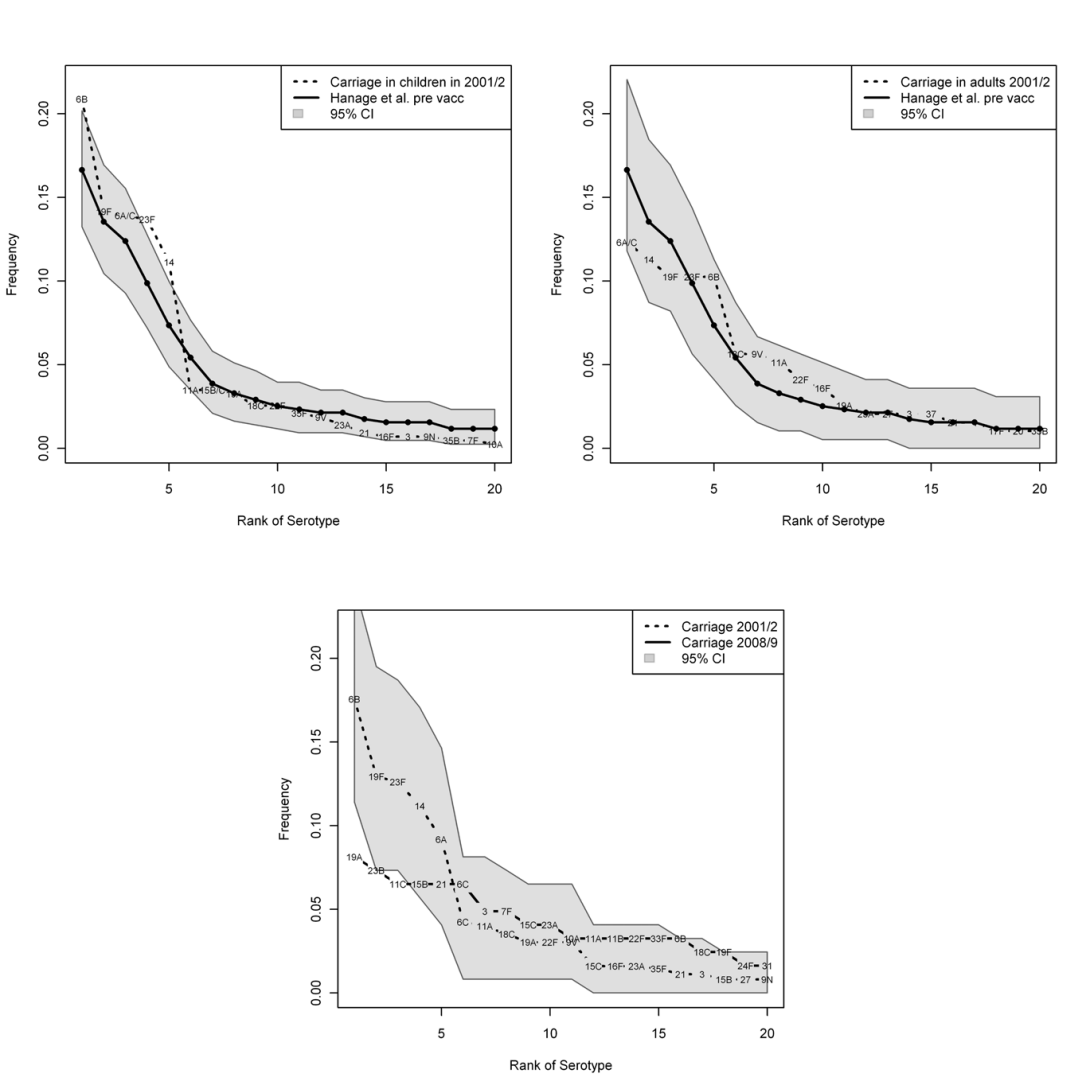
Top: Comparison in ranked-serotype distribution prior to vaccination in children in Massachusetts to our findings in children (left) and adults (right). For comparison with the findings with Hanage and colleagues, we aggregate 6A and 6C to 6A/C and 15B and 15C to 15B/C. Bottom: Changes in ranked serotype distribution in overall carriage in our findings from 2001/2002 to 2008/2009.

Simpson's index of diversity for the 2001/2002 samples was 0.908 95% CI (0.899–0.917); children: 0.891 95% CI (0.878–0.904) and adults: 0.936 95% CI (0.926–0.947). It increased significantly in the 2008/2009 samples to: 0.961 95% CI (0.953–0.969); children: 0.960 95% CI (0.949–0.971) and adults: 0.955 95% CI (0.928–0.982). Furthermore, the ranked frequency distribution of the serotypes, while similar in the prevaccination era in both children and adults in our study compared to children in Massachusetts, changed to become more distinct after vaccination (Figure 1).

Prior to its introduction, PCV7 included types responsible for similar proportions of carriage episodes (62.2%) and disease (55.9%). In 2008/2009 the additional types covered by higher valency vaccines were more prevalent in IPD than carriage, particularly the additional three in PCV10, which comprised 32.6% of IPD but only 4.7% of carried isolates (Table 4).

**Table 4:** Carriage prevalence and IPD incidence in participants less than 60 y caused by serotypes included in PCV7, in PCV10 and not in PCV7, in PCV13 and not in PCV10, and the remaining serotypes.

Serotypes	2008/2009			2001/2002		
	Carriage	Percent	Percent in IPD	Carriage	Percent	Percent in IPD
PCV7	11	(8.7)	15.2	605	(62.2)	55.9
+PCV10	6	(4.7)	32.6	2	(0.2)	10.2
+PCV13	18	(14.2)	15.8	155	(15.9)	8.9
Rest	92	(72.4)	36.4	210	(21.7)	25.0

**Figure 2:**
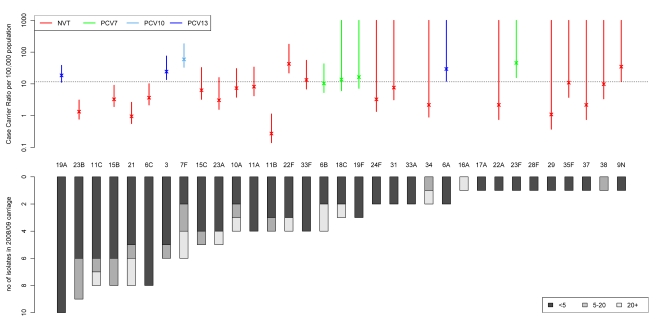
Age-stratified serotype distribution in carriage in 2008/2009 (below, Table S2) and CCR estimated from 2008/2009 carriage and IPD data (above, Table S1). The colour code for the CCR represents that the corresponding serotype is included in PCV7 (green), PCV10 (light blue), PCV13 (dark blue), or is a NVT (red). The dotted line corresponds to the mean CCR for these types. Serotypes 11C, 33A, 16A, 17A, and 28F, although detected in carriage were not found among disease isolates in 2008/2009.

The ranking of carried serotypes by frequency of detection in the post-PCV7 dataset and their associated CCRs as estimated from our 2008/2009 carriage prevalence data are shown in Figure 2. CCR estimates were highly correlated (*p*<0.001, ρ = 0.72) to those from Sleeman and colleagues estimated from carriage incidence [20] and allow to distinguish the more from the less invasive serotypes. From the 15 most prevalent serotypes in carriage in 2008/2009 19A, 3, 7F, and 22F stand out with a generally higher CCR. Despite their high incidence in invasive disease serotypes 1, 8, 12F, 4, and 14 (1.14, 0.58, 0.25, 0.22, 0.14 cases per 100,000 population, respectively, in under 60 y olds in 2008/2009) were not detected in carriage. On the other hand, despite being found in 2008/2009 carriage serotypes, 11C, 16A, 17A, 28F, and 33A were not found in 2008/2009 IPD at all and only caused 0, 1, 0, 1, and 0 cases, respectively, of invasive disease out of over 13,000 isolates serotyped between July 2006 and June 2009.

## Discussion

Our study documents the changes in carried pneumococci following the introduction of PCV7 in England and relates these to concomitant changes in disease in order to assess the invasiveness potential of the serotypes now predominating carriage. This knowledge is essential for understanding replacement pneumococcal disease and provides insight into the likely population impact of higher valency vaccines. As reported elsewhere [13],[14], we found a major reduction in VT carriage in vaccinated children under 5 y, but no overall change in carriage prevalence due to replacement with NVTs. In contrast, there was an overall reduction in IPD in this age group, illustrating that the outcome of the PCV programme as expressed in IPD is determined by the invasiveness potential of the individual NVTs emerging in carriage. Overall carriage prevalence in older unvaccinated siblings and parents was somewhat higher post-PCV7 as found in parents of 2 y olds in a vaccine trial with a 2-dose or a 2+1-dose schedule in the Netherlands [23]. This finding was due to a large increase in NVT and a smaller nonsignificant reduction in VT carriage. However, IPD in these older age groups has not shown an overall increase in the UK [24], indicative of the lower overall invasiveness of the replacing NVTs.

Our study shows that PCV7 provided protection against serotypes that were highly prevalent in both disease and carriage in the UK. The additional serotypes covered by PCV10 and PCV13 are now responsible for a large proportion of invasive disease but were found relatively rarely in carriage (Table 4). While further replacement in pneumococcal carriage is likely to occur after introduction of these higher valency vaccines, our findings suggest that since most of the potential replacement types identified have lower CCRs they will cause less invasive disease. However, serotypes like 22F and especially the ones not found in carriage but present in IPD (e.g., serotype 8 and 12F) could reduce the overall benefits of higher valency vaccines. Interestingly, the three additional serotypes covered by PCV10 had a very low carriage prevalence accounting for <5% of the carried serotypes in 2008/2009 but >30% of IPD cases, whereas the further three serotypes in PCV13 are more similar to the PCV7 serotypes, being similarly prevalent in carriage and disease. While changing to PCV10 has therefore less potential to prevent IPD than PCV13, it may cause fewer perturbations in the nasopharyngeal pneumococcal population. Comparative carriage studies in countries using PCV10 with those using PCV13, or with different PCV coverage of prevalent serotypes before introduction, would be informative to help understand the carriage dynamics underlying serotype replacement. These studies would ideally be repeated cross-sectional studies to monitor alterations in carriage prevalence, which could be linked to changes in serotype-specific IPD in the same population. The latter requires the continued microbiological investigation of suspected cases of invasive disease, including those in fully vaccinated children, in order to document the serotype-specific changes in IPD associated with vaccine-induced changes in carriage.

The diversity of the pneumococcal carriage population in the absence of any external pressure is thought to be relatively stable [22]. If this population is challenged by vaccination with a reduction in the dominance of a few highly prevalent types, the diversity increases and the population takes time to return to the previous level of diversity. Hanage and colleagues suggested methods of assessing these changes: Simpson's index of diversity and the concept of a typical distribution for the ranked frequency of the serotypes [22]. Applying these to our prevaccination carriage data, we see similar diversity in children and slightly higher diversity in adults, although the significance of this difference was not consistent between both methods. However, we found an increase in overall diversity in 2008/2009 as well as in children and in adults (although not significant in adults), consistent with the PCV7-induced changes in the bacterial population still evolving at that time. Evidence for this can also be found in the ongoing changes in non-PCV7 IPD in 2009/2010, prior to introduction of PCV13. These show a continuing increase in the six additional serotypes covered by PCV13 but a decrease in non-PCV13 serotypes in children under 2 y compared with 2008/209 [25]. With the introduction of PCV13 in the UK in March 2010 [26], it will not be possible to evaluate further the longer term impact of PCV7 on carriage and IPD, but it is important to note that PCV7 may continue to have an effect and therefore not all future changes will necessarily be attributable to PCV13.

Recently developed molecular serotyping methods found up to nine times higher proportions of multiple carriage than detectable with standard WHO culturing methods [27]. Using the WHO method we identified one (0.26%) multiple carriage episode in 2008/2009 and four (0.10%) in 2001/2002. Undetected episodes of multiple carriage would result in over estimation of CCRs. However, direct comparison of molecular and conventional serotyping methods have so far only been performed on specimens from developing countries where carriage prevalence is very high [28],[29]. In such settings, molecular methods might reveal more multiple carriage episodes than in countries such as England where carriage prevalence is lower. Furthermore, there is some evidence that detecting multiple serotype carriage is likely to primarily uncover carriage episodes of serotypes previously found to be less prevalent [30]. Therefore we believe that the potential bias introduced by the WHO standard culturing methods would have little impact on our inferences from the CCR, because we focus on the serotypes more common in carriage.

Our study has some limitations. First, the earlier study had a longitudinal design while the recent study was cross-sectional. However, we accounted for multiple testing of individuals in the earlier study as well as differences in age distribution within the age groups, gender, exposure to smoke, and household size by using a GEE, which is designed to fit the parameters of a generalised linear model in the presence of unknown correlation. Second, owing to the lack of power of serotype-specific carriage data in adults, we pooled data of children and adults to derive the CCR, despite different age distributions in the samples for IPD and carriage. Previously reported CCR estimates for children and adults in England and Wales [19] using the carriage data from the earlier study are highly correlated (Figure S1), supporting our use of pooled carriage data from children and adults in the later study. Third, secular changes in serotype distribution in IPD can occur in the absence of vaccination [31], which may be due to alterations in carriage prevalence. With the cross-sectional design of the 2008/2009 study, we were not able to account for these. However, in England the only major secular change in the serotypes causing IPD observed over the last decade has been in serotype 1, which was not detected in either our pre- or post-PCV7 carriage studies. Fourth, invasion is thought to follow shortly after acquisition of carriage rather than being a constant risk throughout the duration of carriage [32]. Thus, a further potential limitation of our study is that we estimate CCRs using carriage prevalence rather than the incidence of new carriage episodes, the latter being derived using prevalence and carriage duration. Few data on serotype-specific duration of carriage are published, and for the serotypes newly emerging after introduction of PCV7, no information is available. Therefore, we used carriage prevalence to get an estimate of the CCRs. Where information on CCRs estimated using carriage incidence was available [20], we found a high correlation with our estimates. Furthermore, our estimates for the CCRs were consistent with those derived from 2001/2002 carriage and IPD (unpublished data), showing that this measure is stable over time. Hence we are confident that our estimates of the CCR can distinguish serotypes with lower invasiveness from those with higher invasiveness.

In conclusion, our study illustrates the value of generating carriage data in parallel with IPD surveillance data to help understand the serotype-specific changes in IPD observed in different epidemiological settings and predict the effect of higher valency vaccines. We provide evidence that the incremental benefit on IPD of the recent switch from PCV7 to PCV13 in the UK, while likely to be substantial, may be somewhat offset by increases in serotypes 8, 12F, and 22F. Such emerging serotypes with high CCRs are potential candidates for inclusion in future conjugate vaccines. More research to elucidate the serotype-specific capsular properties [2],[33] or other factors associated with carriage and invasiveness is needed in order to understand better the likely impact of future conjugate vaccines.

## Supporting Information

Figure S1 Estimated CCR in children and adults from Trotter and colleagues [19]. The grey lines represent the confidence bounds. Spearman's rank test for correlation: *p* = 0.01, ρ = 0.62.
(TIF)

Table S1 Estimated CCR for all serotypes found in carriage in 2008/2009 with the corresponding number of isolates found in carriage and in IPD in the under 60-y-old population. Some serotypes were found in IPD but not in carriage: sertoype 1 (387 isolates), 8 (196), 12F (83), 4 (74), 9V (58), 14 (48), 5 (23), 20 (21), 15A (12), 17F (10), 16F (10), 35B (6), 27 (6), 13 (4), 28A (3), 12B (3), and one each of 9L, 7C, 7B, 7A, 7, 6, 35A, 28, 18A, 10F. *A total of 81 isolates of 6A/6C were found in IPD of which one-third was assumed to be 6C and the rest 6A.
(DOC)

Table S2 Number of isolates found in carriage in 2001/2002 and 2008/2009. In 2001/2002 carriage of a second serotype was detected in four isolates; serotypes 22F (1 isolate), 3 (2), and 6B (1) were found. In 2008/2009 additional carriage of serotype 21 was detected once. ^*^6A and 6C were not distinguished in 2001/2002.
(DOC)

## Abbreviations

CCR: case∶carrier ratio
GEE: Generalized Estimating Equation
IPD: invasive pneumococcal disease
NVT: non–vaccine type
PCV7: 7-valent pneumococcal conjugate vaccine
VT: vaccine type
