# Supplementary figures and images for: Effect of Pneumococcal Conjugate Vaccination on Serotype-Specific Carriage and Invasive Disease in England: A Cross-Sectional Study
Source: PLoS Med. 2011 Apr 5;8(4):e1001017. doi: 10.1371/journal.pmed.1001017 (PMC3071372; doi:10.1371/journal.pmed.1001017)

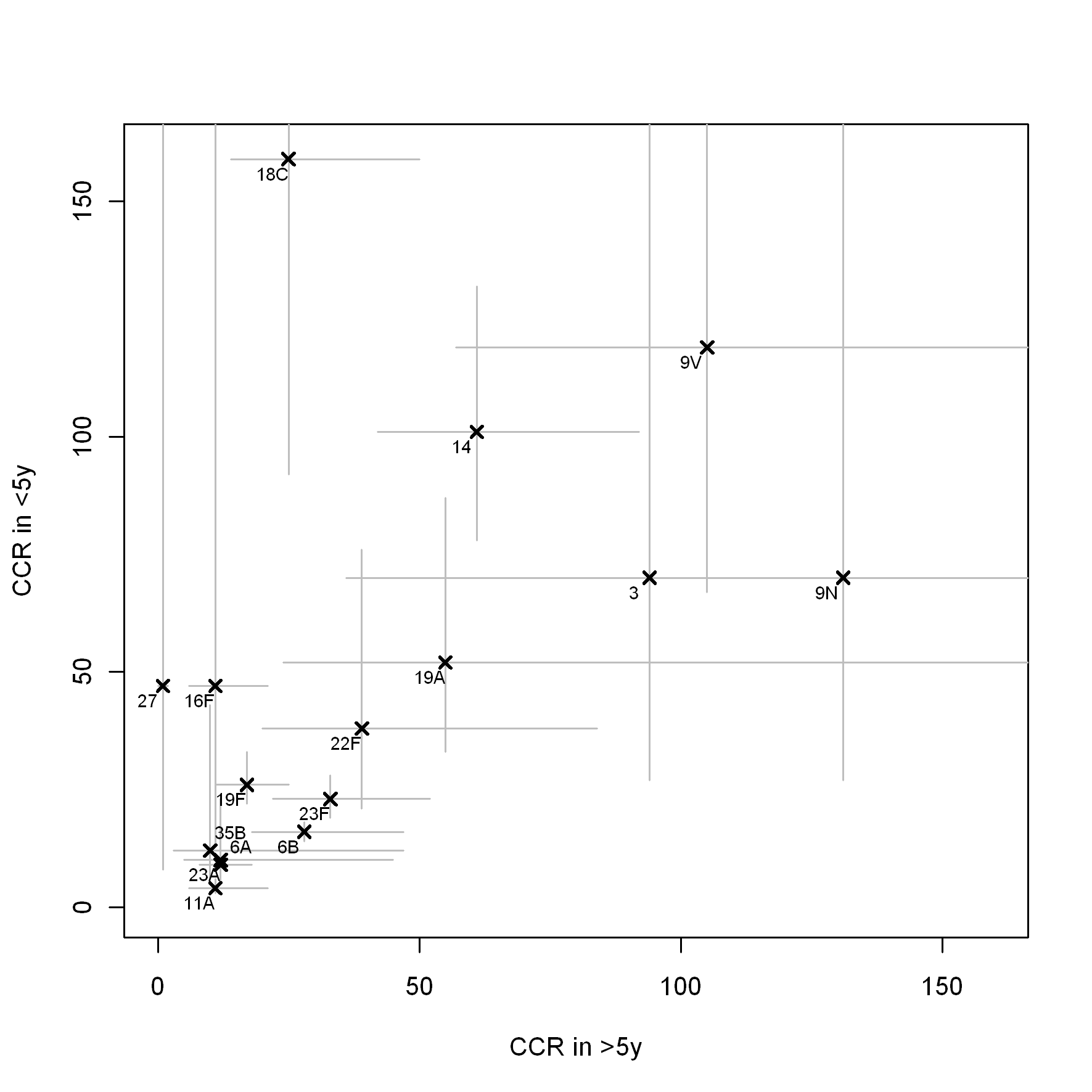

Supplement: Figure S1 — Estimated CCR in children and adults from Trotter and colleagues [19]. The grey lines represent the confidence bounds. Spearman's rank test for correlation: p = 0.01, ρ = 0.62. (TIF) [file pmed.1001017.s001.tif]
