# Supplementary material for: Effect of Pneumococcal Conjugate Vaccination on Serotype-Specific Carriage and Invasive Disease in England: A Cross-Sectional Study
Source: PLoS Med. 2011 Apr 5;8(4):e1001017. doi: 10.1371/journal.pmed.1001017 (PMC3071372; doi:10.1371/journal.pmed.1001017)
Supplement: Table S1 — Estimated CCR for all serotypes found in carriage in 2008/2009 with the corresponding number of isolates found in carriage and in IPD in the under 60-y-old population. Some serotypes were found in IPD but not in carriage: sertoype 1 (387 isolates), 8 (196), 12F (83), 4 (74), 9V (58), 14 (48), 5 (23), 20 (21), 15A (12), 17F (10), 16F (10), 35B (6), 27 (6), 13 (4), 28A (3), 12B (3), and one each of 9L, 7C, 7B, 7A, 7, 6, 35A, 28, 18A, 10F. *A total of 81 isolates of 6A/6C were found in IPD of which one-third was assumed to be 6C and the rest 6A. (DOC) [file pmed.1001017.s002.doc]

|  | Carriage 2008/09 | IPD 2008/09 | Cases per 100,000 carriers |
| --- | --- | --- | --- |
| 19A | 10 | 170 | 18.6 ( 11.3 - 38.3 ) |
| 23B | 9 | 11 | 1.3 ( 0.8 - 3.1 ) |
| 11C | 8 | 0 | 0 ( 0 - 0 ) |
| 15B | 8 | 24 | 3.3 ( 1.9 - 9 ) |
| 21 | 8 | 7 | 1 ( 0.6 - 2.6 ) |
| 6C^*^ | 8 |  | 3.7 ( 2.2 - 10.1 ) |
| 3 | 6 | 134 | 24.4 ( 13.7 - 75.6 ) |
| 7F | 6 | 326 | 59.3 ( 33.4 - 183.8 ) |
| 15C | 5 | 29 | 6.3 ( 3.3 - 32.7 ) |
| 23A | 5 | 14 | 3.1 ( 1.6 - 15.8 ) |
| 10A | 4 | 27 | 7.4 ( 3.8 - 30.4 ) |
| 11A | 4 | 30 | 8.2 ( 4.2 - 33.8 ) |
| 11B | 4 | 1 | 0.3 ( 0.1 - 1.1 ) |
| 22F | 4 | 157 | 42.9 ( 22.1 - 177 ) |
| 33F | 4 | 49 | 13.4 ( 6.9 - 55.3 ) |
| 6B | 4 | 38 | 10.4 ( 5.4 - 42.9 ) |
| 18C | 3 | 38 | 13.8 ( 6.1 - Inf ) |
| 19F | 3 | 45 | 16.4 ( 7.2 - Inf ) |
| 24F | 2 | 6 | 3.3 ( 1.4 - Inf ) |
| 31 | 2 | 14 | 7.6 ( 3.2 - Inf ) |
| 33A | 2 | 0 | 0 ( 0 - NaN ) |
| 34 | 2 | 4 | 2.2 ( 0.9 - Inf ) |
| 6A^*^ | 2 |  | 29.5 ( 12.2 - Inf ) |
| 16A | 1 | 0 | 0 ( 0 - NaN ) |
| 17A | 1 | 0 | 0 ( 0 - NaN ) |
| 22A | 1 | 2 | 2.2 ( 0.8 - Inf ) |
| 23F | 1 | 42 | 45.9 ( 15.8 - Inf ) |
| 28F | 1 | 0 | 0 ( 0 - NaN ) |
| 29 | 1 | 1 | 1.1 ( 0.4 - Inf ) |
| 35F | 1 | 10 | 10.9 ( 3.8 - Inf ) |
| 37 | 1 | 2 | 2.2 ( 0.8 - Inf ) |
| 38 | 1 | 9 | 9.8 ( 3.4 - Inf ) |
| 9N | 1 | 32 | 34.9 ( 12 - Inf ) |
| Not typed | 4 | 546 |  |
