# Supplementary material for: Effect of Pneumococcal Conjugate Vaccination on Serotype-Specific Carriage and Invasive Disease in England: A Cross-Sectional Study
Source: PLoS Med. 2011 Apr 5;8(4):e1001017. doi: 10.1371/journal.pmed.1001017 (PMC3071372; doi:10.1371/journal.pmed.1001017)
Supplement: Table S2 — Number of isolates found in carriage in 2001/2002 and 2008/2009. In 2001/2002 carriage of a second serotype was detected in four isolates; serotypes 22F (1 isolate), 3 (2), and 6B (1) were found. In 2008/2009 additional carriage of serotype 21 was detected once. *6A and 6C were not distinguished in 2001/2002. (DOC) [file pmed.1001017.s003.doc]

|  | **2008/09** | | | | **2001/02** | | | |
| --- | --- | --- | --- | --- | --- | --- | --- | --- |
|  | **<5y** | **5-20y** | **≥20y** | **All** | **<5y** | **5-20y** | **≥20y** | **All** |
| 10A | 2 | 1 | 1 | 4 | 1 | 0 | 1 | 2 |
| 11A | 4 | 0 | 0 | 4 | 27 | 8 | 3 | 38 |
| 11B | 3 | 0 | 1 | 4 | 0 | 0 | 0 | 0 |
| 11C | 6 | 1 | 1 | 8 | 0 | 0 | 0 | 0 |
| 14 | 0 | 0 | 0 | 0 | 64 | 24 | 5 | 93 |
| 15B | 6 | 0 | 2 | 8 | 5 | 0 | 0 | 5 |
| 15C | 4 | 0 | 1 | 5 | 14 | 0 | 0 | 14 |
| 16A | 0 | 1 | 0 | 1 | 0 | 0 | 0 | 0 |
| 16F | 0 | 0 | 0 | 0 | 3 | 1 | 9 | 13 |
| 17A | 1 | 0 | 0 | 1 | 0 | 0 | 0 | 0 |
| 17F | 0 | 0 | 0 | 0 | 0 | 0 | 2 | 2 |
| 18B | 0 | 0 | 0 | 0 | 2 | 0 | 0 | 2 |
| 18C | 2 | 1 | 0 | 3 | 13 | 3 | 9 | 25 |
| 19A | 10 | 0 | 0 | 10 | 18 | 3 | 2 | 23 |
| 19D | 0 | 0 | 0 | 0 | 1 | 0 | 0 | 1 |
| 19F | 3 | 0 | 0 | 3 | 100 | 24 | 3 | 127 |
| 20 | 0 | 0 | 0 | 0 | 0 | 1 | 1 | 2 |
| 21 | 5 | 2 | 1 | 8 | 6 | 0 | 4 | 10 |
| 22A | 1 | 0 | 0 | 1 | 0 | 0 | 0 | 0 |
| 22F | 3 | 1 | 0 | 4 | 14 | 7 | 2 | 23 |
| 23A | 4 | 1 | 0 | 5 | 9 | 5 | 0 | 14 |
| 23B | 6 | 0 | 3 | 9 | 0 | 0 | 0 | 0 |
| 23F | 1 | 0 | 0 | 1 | 97 | 10 | 13 | 120 |
| 24F | 2 | 0 | 0 | 2 | 0 | 0 | 0 | 0 |
| 27 | 0 | 0 | 0 | 0 | 1 | 3 | 3 | 7 |
| 28F | 1 | 0 | 0 | 1 | 0 | 0 | 0 | 0 |
| 29 | 1 | 0 | 0 | 1 | 0 | 0 | 0 | 0 |
| 3 | 5 | 0 | 1 | 6 | 3 | 7 | 0 | 10 |
| 31 | 2 | 0 | 0 | 2 | 1 | 1 | 0 | 2 |
| 33A | 2 | 0 | 0 | 2 | 0 | 0 | 0 | 0 |
| 33F | 4 | 0 | 0 | 4 | 1 | 0 | 0 | 1 |
| 34 | 0 | 1 | 1 | 2 | 0 | 0 | 1 | 1 |
| 35A | 0 | 0 | 0 | 0 | 0 | 0 | 1 | 1 |
| 35B | 0 | 0 | 0 | 0 | 4 | 2 | 1 | 7 |
| 35C | 0 | 0 | 0 | 0 | 0 | 0 | 1 | 1 |
| 35F | 1 | 0 | 0 | 1 | 16 | 0 | 0 | 16 |
| 37 | 1 | 0 | 0 | 1 | 0 | 0 | 5 | 5 |
| 38 | 0 | 0 | 1 | 1 | 1 | 0 | 0 | 1 |
| 4 | 0 | 0 | 0 | 0 | 0 | 0 | 3 | 3 |
| 6A^*^ | 2 | 0 | 0 | 2 | 94 | 21 | 7 | 122 |
| 6B | 2 | 2 | 0 | 4 | 193 | 11 | 10 | 214 |
| 6C^*^ | 8 | 0 | 0 | 8 | 0 | 0 | 0 | 0 |
| 7F | 2 | 2 | 2 | 6 | 2 | 0 | 0 | 2 |
| 8 | 0 | 0 | 0 | 0 | 0 | 2 | 0 | 2 |
| 9N | 0 | 0 | 0 | 0 | 5 | 2 | 0 | 7 |
| 9N | 1 | 0 | 0 | 1 | 12 | 7 | 4 | 23 |
| NTR | 3 | 0 | 1 | 4 | 20 | 5 | 8 | 33 |
